# 562. Diagnostic accuracy comparison of the Biofire Filmarray pneumonia panel versus routine diagnostic methods for diagnosis of ventilator associated pneumonia: a real life study

**DOI:** 10.1093/ofid/ofad500.631

**Published:** 2023-11-27

**Authors:** Alejandra García-Martínez, Leticia Rojo-Gutiérrez, Sergio Llanes-Somellera, Daniel Aguilar-Zapata

**Affiliations:** Hospital Médica Sur, Mexico City, Distrito Federal, Mexico; Hospital Médica Sur, Mexico City, Distrito Federal, Mexico; Universidad Valle de México, Mexico City, Distrito Federal, Mexico; Hospital Medica Sur, Mexico City, Mexico City, Mexico

## Abstract

**Background:**

In Mexico, the Hospital Epidemiological Surveillance Network previously the COVID-19 pandemic, reported a total of 61,969 health care associated infections with an incidence rate of 4.7 per 100 discharges, 20.7% of which were categorized as ventilator associated pneumonia.

Biofire Filmarray pneumonia panel has been accepted as a tool for early and rapid diagnostic of pneumonia with recognition of several hospital acquired pathogens including resistant genes. However, there are some mismatch in the multiplex PCR detection and the culture isolation.

**Methods:**

An observational, comparative, retrospective, non-randomized, cross-sectional, open study was performed using files from patients diagnosed with ventilator associated pneumonia who underwent diagnostic testing on culture and PCR multiplex FilmArray pneumonia panel to determine diagnostic accuracy.

Intensive care unit files from August 2021 to February 2022 with ventilator associated pneumonia with airway samples for Filmarray pneumonia panel and cultures. Evaluation of the used diagnostic tests was carried out using: sensitivity, specificity, positive predictive value and negative predictive value.

**Results:**

55 patients were included, each of them with both test. 72% of the study population were males, with a mean age of 61 years. 76% of the population had severe COVID-19.

*E.Coli had* 100% sentitivity and 96% specificity , *P. aeruginosa 100% s*entitivity and 88% specificity, *E.Cloaca*e 100% sentitivity and 91% specificity, and *S. aureus* had 100% sentitivity and 89% specificity on Biofire Filmarray pneumonia panel. While *K. pneumoniae* had 50% sensitivity and 89% specificity. NPV was 98% for *K. pneumonia*e, and 100% for *E. coli, P. aeruginosa, E. cloacae*, and *S. aureus.*

*S.maltophilia* was isolated in 3 culture samples and the Filmarray pneumonia panel was reported negative because lacking of testing this microorganism, as well as 1 *A. fumigatus* isolation.

Samples for cultures and Biofire FilmArray pneumonia panel

**Figure:**
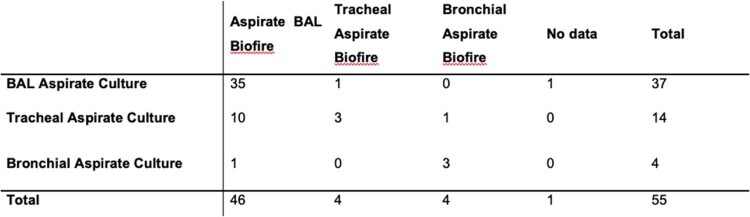

The different samples that were included from 55 patients in this study correlated with pneumonia panel and conventional cultures.

PCR Biofire FilmArray pneumonia panel diagnostic study evaluation

**Figure:**
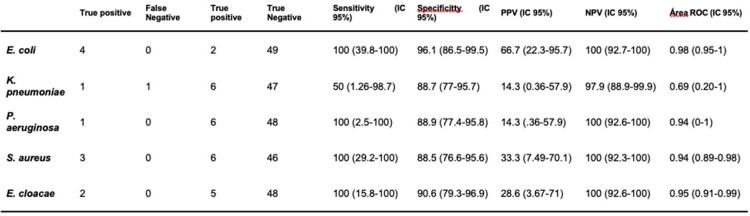

Each of the microorganism that had correlation between Filmarray pneumonia panel compared with conventional cultures

**Conclusion:**

Biofire Filmarray pneumonia panel provides early and accurate information on pathogens causing ventilator associated pneumonia. However it cannot replace conventional culture because of lacking of some microorganism detection. It provides a useful tool for early decision-making in treatment.

**Disclosures:**

**All Authors**: No reported disclosures

